# Electrical Stimulation‐Induced Muscle Damage Alters Hippocampal BDNF Signaling

**DOI:** 10.1111/ejn.70235

**Published:** 2025-09-17

**Authors:** Julien Wirtz, Rémi Chaney, Marina Cefis, Alexandre Méloux, Yuan Wang, Stéphanie Lemaire, Aurore Quirié, Julien Delezie, Gilles Gouspillou, Anne Prigent‐Tessier, Philippe Garnier

**Affiliations:** ^1^ Université Bourgogne Europe, Inserm, CAPS UMR1093 Dijon France; ^2^ Département des Sciences de l'Activité Physique, Faculté des Sciences Université du Québec à Montréal Montréal Quebec Canada; ^3^ Département Génie Biologique Institut Universitaire de Technologie Dijon France; ^4^ Centre Hospitalier Universitaire, Service de Biochimie Spécialisée Dijon France

**Keywords:** BDNF, ES (electrical stimulation), FNDC5/Irisin, hippocampus, inflammation, muscle injury, neuroplasticity

## Abstract

This study investigates whether electrical stimulation (ES) could mimic traditional exercise in enhancing brain‐derived neurotrophic factor (BDNF)‐dependent neuroplasticity via muscle‐brain communication, specifically through the fibronectin type III domain‐containing protein 5 (FNDC5)/Irisin pathway. Male Wistar rats received transcutaneous ES targeting the lumbar nerve roots to induce hindlimb muscle contractions for 30 min daily over seven consecutive days. Blood and tissue samples were collected for biochemical, histological, and molecular analyses 1 day after the final session. Our findings reveal that ES disrupted BDNF signaling in the hippocampus, reducing synaptic protein expression. At the muscular level, ES caused significant damage, particularly in the soleus muscle, accompanied by muscle satellite cell (MuSC) activation, proliferation, and differentiation. Notably, ES increased FNDC5 expression in injured muscles, but this was associated with MuSC activation rather than humoral communication between muscle and brain. Moreover, a positive correlation was observed between the pro‐inflammatory state of the injured muscles and hippocampal glucocorticoid receptor activation, as an indicator of stress, which was linked to impaired BDNF signaling. These results suggest two key conclusions: (1) Increased FNDC5 expression in damaged muscle fibers primarily reflects local repair mechanisms rather than a beneficial humoral dialogue, and (2) ES protocols that induce muscle injury can negatively impact BDNF‐dependent plasticity by triggering maladaptive muscle‐brain interactions. These findings highlight the importance of optimizing muscle stimulation protocols to minimize muscle damage, particularly when applied to individuals unable to engage in conventional physical activity or suffering from muscle weakness.

## Graphical abstract

Electrical stimulation (ES) causes muscle damage, local inflammation (↑IL‑1β, CD68), muscular satellite cells (MuSCs) differentiation (↑PAX7, MyoD1), and upregulation of FNDC5/Irisin attributable in part to MuSCs. While FNDC5/Irisin normally mediates beneficial muscle‑to‑brain dialogue, ES‑induced inflammation‐linked signal generates maladaptive cues, resulting in hippocampal neuroplasticity impairment.

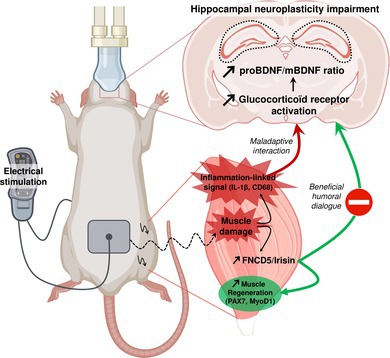

**Abbreviations**
BDNF: brain‐derived neurotrophic factor
ES: electrical stimulation
FNDC5: fibronectin type III domain‐containing protein 5
GAP43: growth‐associated protein 43
GAS: gastrocnemius muscle
IL‐1β: Interleukin‐1 beta
JNK: c‐Jun N‐terminal kinase
MuSC: muscle satellite cell
MyoD1: myoblast determination protein 1
NMES: neuromuscular electrical stimulation
Pax‐7: paired box protein 7
PSD95: postsynaptic density protein 95
SOL: soleus muscle
SYP: synaptophysin
TrkB: tropomyosin receptor kinase B

## 1 Introduction

Skeletal muscles, representing about 40% of total body mass tissue, are crucial for locomotion, posture, respiration, thermogenesis, and metabolic homeostasis (Beausejour et al. 2024). Over the past two decades, research has uncovered their significant endocrine function through the secretion of myokines (Severinsen and Pedersen 2020), which play essential roles in supporting brain homeostasis and function (Delezie and Handschin 2018; Pedersen 2019). Among them, the myokine irisin, a cleaved and secreted product of fibronectin type III domain‐containing protein 5 (FNDC5), has gained considerable attention for its neuromodulatory effects (Islam et al. 2021; Wrann et al. 2013). Studies have shown that muscle‐secreted irisin crosses the blood–brain barrier (BBB) and upregulates brain‐derived neurotrophic factor (BDNF), a crucial neurotrophin for neuronal survival and synaptic plasticity (Lu et al. 2013; C. Wang, Kavalali, and Monteggia 2022). Conversely, muscle dysfunction such as in Duchenne muscular dystrophy (Rae and O'Malley 2016), myotonic dystrophy (Modoni et al. 2004), or sarcopenia (Tessier et al. 2022) is associated with cognitive impairment and the progression of Alzheimer's disease (Brisendine and Drake 2023). Moreover, musculoskeletal injuries can negatively impact cognitive function in both healthy and athletic individuals (Hutchison et al. 2011). Animal models of muscle atrophy and injury, i.e., using the Swedish mutant APP mouse model (Pan et al. 2022) or freezing‐induced muscle injuries (Guéniot et al. 2020), demonstrate similar cognitive impairments. These findings collectively underscore the role of skeletal muscles as critical endocrine organs and suggest that muscle health can establish either a beneficial or detrimental dialogue with the brain.

Beyond providing benefits to metabolic and cardiovascular health, exercise is widely recognized as the most effective nonpharmacological strategy to enhance brain health (Mandolesi et al. 2018; Sujkowski et al. 2022). At the cerebral level, BDNF plays a pivotal role in mediating these central benefits (Vaynman et al. 2004). Initially synthesized as proBDNF, this neurotrophin undergoes cleavage to form mature BDNF (mBDNF) (R. Jones 2004), each interacting with distinct plasma membrane receptors, respectively, p75^NTR^ and TrkB. This processing is critical since the activation of TrkB by mBDNF is associated with beneficial effects on neuronal survival and synaptic plasticity, whereas binding of proBDNF to p75^NTR^ triggers pro‐apoptotic pathways and impairs synaptic transmission (Lu et al. 2005). Exercise‐induced cerebral BDNF production is thought to result from three main mechanisms, namely, the increase in neuronal expression through activity‐dependent mechanisms, the increase in cerebral blood flow (CBF, also called the hemodynamic hypothesis), and the release of myokines derived from contracting muscles (Cefis et al. 2023).

Despite evidence and prevailing recommendations underscoring the potential of exercise to enhance cerebral function, many individuals encounter barriers to participating in active physical activity. For these individuals, neuromuscular electrical stimulation (NMES) has emerged as a potential alternative to traditional exercise (S. Jones et al. 2016). Recent research suggests that NMES can mimic traditional exercise benefits for executive function in humans and BDNF signaling in rats (Chaney et al. 2024). Although localized NMES of individual muscles (e.g., quadriceps or gastrocnemius) does not significantly increase circulating irisin levels (Maekawa et al. 2018; Szabó et al. 2023), whole‐body NMES sessions have been shown to elevate blood irisin levels (Ghalamsiah and Nourshahi 2023) despite the potential for severe muscle damage and rhabdomyolysis (Kästner et al. 2015). Therefore, the effect of intense or extended NMES on muscle‐brain communication, in particular on the expression of central BDNF, remains unexplored.

In this context, we aimed to investigate the effects of chronic daily electrical stimulation (ES) on a large muscle mass on muscle‐brain communication, focusing on the FNDC5/Irisin pathway and BDNF hippocampal signaling. ES was applied chronically to the lumbar nerve roots to induce simultaneous contraction of both hindlimbs in Wistar male rats. We hypothesized that chronic ES by recruiting all the muscles of the lower limbs would stimulate FNDC5 synthesis and irisin release, potentially promoting hippocampal BDNF expression and downstream signaling. Several key aspects were assessed, including hippocampal BDNF expression and related signaling, muscle‐derived irisin expression as well as muscle integrity and regeneration, using molecular, biochemical, and histological analyses. This comprehensive approach allowed us to examine the complex interplay between ES‐induced muscle changes and hippocampal signaling, providing insights into the risk‐benefit ratio of ES as an exercise alternative.

## 2 Methods

### 2.1 Animals

The experiments were conducted in accordance with guidelines set forth by the French Department of Agriculture (licence 21‐CAE‐102) and approved by the local ethics committee (Ethics Committee of Animal Experimentation, Dijon, registration number 105). The study involved 10 8‐week‐old male Wistar rats, purchased from Janvier Labs (Le Genest Saint Isle, France). The rats were housed in standard conditions with a 12‐h light/dark cycle and provided ad libitum access to food and water.

### 2.2 ES

The ES protocol has been described previously in detail (Chaney et al. 2022). Briefly, rats were first anesthetized using isoflurane (4% for induction in a clear chamber, followed by 2% for maintenance via a standard rat nose mask). During the anesthesia, the body temperature of the rats was maintained at approximately 37°C using a heating pad.

Electrodes of 7‐mm diameter (Contrôle Graphique Medical, Brie‐Compte‐Robert, France) were connected to an electrical stimulator (DS7AH, Digitimer, Hertfordshire, United Kingdom) controlled by TIDA software (Heka Elektronik, Lambrecht/Pfalz, Germany). All rats were shaved on their back and abdomen. Electrodes were placed with the anode on the abdomen and the cathode above the lumbar vertebra (L6). This electrode placement was selected to enable activation of a broad muscle mass across all hindlimb muscles, as evidenced by the contraction of both flexor and extensor muscles.

Stimulation consisted of a 100‐Hz biphasic current with 200‐μs pulses, alternating 6 s ON and 3 s OFF over 30 min. The current intensity, initially set at 5 times the motor threshold—defined as the minimum intensity required to elicit hindlimb contractions (7 mA), was adjusted up to 70 mA to maintain the desired torque output, which was not precisely measured but only visually estimated by producing a consistent and reproducible joint movement in terms of range of motion and muscle response.

After a 7‐day familiarization period with the experimenters, rats received daily 30‐min stimulation sessions from 8 to 11 a.m. for 7 consecutive days. Twenty‐four hours after the final session, the animals were euthanized by transcardial perfusion with physiological saline under isoflurane anesthesia. The SOL and medial part of the GAS muscles, along with the hippocampus, were collected and immediately frozen at −80°C for subsequent biochemical analyses by Western blot or immediately placed in a 4% paraformaldehyde (PFA) bath for further histological analyses. Blood samples were also collected via intracardiac puncture, placed in light‐protected collection tubes for 45 min, centrifuged at 2000 *g* for 15 min, and then frozen at −80°C for ELISA analyses.

The body weight of the animals was monitored throughout the experimental procedure (Days 1 to 7), and no significant difference between the ES and SHAM groups was observed. Similarly, SOL and GAS muscle mass were comparable between the two groups (Figure S1A).

### 2.3 Western Blotting

Proteins from skeletal muscle and hippocampal tissues were homogenized in a lysis buffer (100‐mM Tris Base, 150‐mM NaCl, 1‐mM EGTA, 1% triton X100, protease and phosphatase inhibitors, pH 7,4) using a tenfold volume ratio, followed by sonication for 15 s. Subsequently, the samples were centrifuged at 15,000 *g* for 15 min at 4°C. The supernatants were collected and immediately frozen at −80°C. Protein concentration in the samples was determined using the Lowry method (Lowry Pierce, ThermoFisher).

Samples were mixed with 1X Laemmli buffer (Tris 125 mM, SDS 4%, Glycerol 20%, Bromophenol Blue 0.01%); then, equal amounts of protein samples were separated by SDS‐PAGE electrophoresis on polyacrylamide gels (TGX Stain‐Free FastCast Acrylamide Kit, 7.5%, 1610181 or 12%, 161‐0185, Bio‐Rad). Gels containing proteins were activated to UV light with Chemidoc imaging systems (12003153, Bio‐Rad) using the “Stain free gel activation (45s)” program. Thereafter, proteins were transferred to a 0.2‐μm nitrocellulose or PVDF membrane (1620112, 1620177, Biorad) using Turbo Transblot transfer system (1704150, Biorad). Membranes were blocked with 5% nonfat milk for 1 h at room temperature (RT), and then probed overnight at 4°C with primary antibodies. Table 1 provides the complete list of antibodies used for Western blotting. The following day, membranes were incubated with corresponding HRP‐conjugated secondary antibody for 1 h at RT. Membranes were revealed using enhanced chemiluminescence substrate (Biorad, Clarity ECL substrate, 170‐5060) and scanned with ChemiDoc imaging system (Biorad). Band intensities were analyzed using ImageLab software (Version 6.0.1, Biorad) and standardized on total protein.

**TABLE 1:** List of antibodies used for Western blotting.

Target protein	Dilution	Reference
**Akt (pan)**	1/3000	*Rabbit anti‐Akt (pan) antibody (#4691) Cell Signaling Technology*
**proBDNF/mBDNF**	1/3000	*Rabbit anti‐BDNF antibody (ab108319) Abcam*
**Caspase 3**	1/3000	*Rabbit anti‐Caspase‐3 antibody (#9665) Cell Signaling Technology*
**c‐Fos**	1/3000	*Rabbit anti‐cfos antibody (GTX129846) GeneTex*
**Cyt c**	1/3000	*Rabbit anti‐Cytochrome c antibody (ab90529) Abcam*
**Erk1/2**	1/3000	*Rabbit anti‐p44/42 MAPK (Erk1/2) antibody (#9102) Cell Signaling Technology*
**FNDC5**	1/3000	*Rabbit anti‐FNDC5 antibody (ab174833) Abcam*
**GAP43**	1/3000	*Rabbit anti‐GAP43 antibody (#8945) Cell Signaling Technology*
**GFAP**	1/3000	*Mouse anti‐GFAP antibody (G3893) Sigma Aldrich*
**Iba1**	1/3000	*Goat anti‐Iba1 antibody (ab5076) Abcam*
**IL1β**	1/3000	*Rabbit anti‐IL1β antibody (GTX74034) GeneTex*
**JNK**	1/3000	*Rabbit anti‐SAPK/JNK antibody (#9252) Cell Signaling Technology*
**MyoD1**	1/3000	*Mouse anti‐MyoD1 antibody (ab16148) Abcam*
**p75NTR (NGFR)**	1/3000	*Rabbit anti‐NGFR antibody (FNab06091) Fine Test*
**P‐Akt (S473)**	1/3000	*Rabbit anti‐Phospho‐Akt (S473) antibody (#4060) Cell Signaling Technology*
**panAkt**	1/3000	*Rabbit anti‐Akt (pan) antibody (#4691) Cell Signaling Technology*
**PAX7**	1/3000	*Mouse anti‐PAX7 antibody (ab199010) Abcam*
**P‐eNOS (S1177)**	1/3000	*Mouse anti‐eNOS (S1177) antibody (612393) BD Biosciences*
**eNOS**	1/3000	*Mouse anti‐eNOS antibody (610297) BD Biosciences*
**P‐Erk1/2**	1/3000	*Rabbit anti‐Phospho‐p44/42 MAPK (Erk1/2) (T202/Y204) antibody (#4370) Cell Signaling Technology*
**P‐FAK (Y397)**	1/3000	*Rabbit anti‐Phospho‐FAK (Y397) antibody (#3283) Cell Signaling Technology*
**P‐Glucocorticoid Receptor**	1/3000	*Rabbit anti‐Phospho‐Glucocorticoid Receptor (S211) antibody (#4161) Cell Signaling Technology*
**P‐JNK**	1/3000	*Rabbit anti‐Phospho‐SAPK/JNK (T183/Y185) antibody (#4668) Cell Signaling Technology*
**PSD95**	1/3000	*Rabbit anti‐PSD95 antibody (#3450) Cell Signaling Technology*
**P‐TrkB (Y816)**	1/3000	*Rabbit anti‐TrkB (Y816) antibody (ABN1381) Sigma‐Aldrich*
**Synaptophysin**	1/3000	*Rabbit anti‐synaptophysin (Epredia RB1461‐P1) ThermoFisher*
**TrkB‐FL**	1/3000	*Mouse anti‐TrkB antibody (610102) BD Biosciences*
**HRP‐conjugated secondary anti‐rabbit**	1/20000	*HRP‐conjugated secondary anti‐rabbit (111‐035‐144) Jackson Immunoresearch*
**HRP‐conjugated secondary anti‐mouse**	1/20000	*HRP‐conjugated secondary anti‐mouse (115‐035‐166) Jackson Immunoresearch*
**HRP‐conjugated secondary anti‐goat**	1/10000	*HRP‐conjugated secondary anti‐goat (ab6741) Abcam*

### 2.4 Blood ELISA

Irisin levels in serum were determined with a commercial ELISA kit (EK‐067‐29, Phoenix Pharmaceuticals, Schiltigheim, France). According to the manufacturer's instructions, samples were diluted at 1/10, and the limit of sensitivity was set at 1.29 ng·mL^−1^. All samples were assayed in duplicate, and a positive control validated the experimental conditions. Plasma levels of IL‐1β were measured using Milliplex magnetic bead panel kits (Merck Millipore, Burlingame, CA, US) that were analyzed using a Luminex MAGPIX system (Luminex Corporation; Houston, TX) and Milliplex Analyst software (Millipore; St. Charles, MO). The limit of detection was 1.22 pg·mL^−1^.

### 2.5 Corticosterone Measurement

Corticosterone levels were measured using a commercially available kit (MassChrom, Chromsystems, Germany) following the manufacturer's instructions. Briefly, 500 μL of plasma diluted to 1/5 was mixed with an internal standard and loaded onto an SPE plate with an extraction buffer via centrifugation. After two washing steps and elution in 500 μL of elution buffer, the extracts were evaporated to dryness and reconstituted in 100 μL of reconstitution buffer. Chromatography was performed with an Acquity UPLC system (Waters Corporation) coupled to a Xevo TQS micro tandem mass spectrometer (Waters Corporation).

### 2.6 Muscle Histology

SOL and GAS muscles were fixed during 48 h in 4% PFA solution (9713, VWR) and dehydrated in consecutive alcohol baths: ethanol 70° (1 × 30 min), ethanol 95° (3 × 60 min), and ethanol 100° (3 × 60 min). Samples were then incubated in methylcyclohexane (3 × 60 min) and embedded in paraffin (3 × 2 h). Each step was carried out in a dehydration automaton (ASP300, Leica). Using a microtome (RM2245, Leica), 5‐μm muscle sections were cut, collected on SuperFrost Plus slides (ThermoFisher) and dried overnight at 45°C. Sections were deparaffinized and rehydrated through successive baths of xylene, ethanol, and deionized water. Subsequently, muscle sections were stained using baths of hematoxylin (Harris Hematoxylin, Leica 3801562E, Wetzlar, Germany) and Y‐eosin (Eosin Y, Leica 3801601E) or Sirius red in 1% picric acid solution (B21693.14, ThermoFisher) and were cover‐slipped using Eukitt mounting media (ORSAtec GmbH). Slides were imaged using a brightfield microscope (Axioscop Imager.M2, Zeiss, Oberkochen, Germany). Fibrosis quantification analysis was automatically performed using the ImageJ analysis software (NIH, Bethesda, Maryland, USA). Whole muscle slides were segmented using the minimum dark automatic thresholding method on the channel of interest after black/white balance enhancement, and the total area in μm^2^ was extracted using the metadata of each image. The integrated intensity was measured in the combined ROIs on the raw image.

### 2.7 Immunohistofluorescence Labeling

After deparaffination and rehydration, sections were unmasked with an enzymatic solution (10‐μg·mL^−1^ proteinase K in PBS) for 10 min at 37°C. After three washes (5 min) in phosphate buffer solution (PBS), muscle sections were blocked with Tween buffer solution (TBS) containing 10% goat serum and 0.5% Tween for 1 h at RT. Slides were incubated overnight at 4°C with primary antibodies (Table 2) diluted in blocking buffer. Slides were then washed three times in PBS (5 min each) and incubated with secondary antibody (Table 2) solution for 1 h at RT. Fluorescently conjugated secondary antibodies were diluted in blocking buffer and applied for 1 h at RT in the dark, followed by three washes in PBS. Finally, slides were mounted with fluoro‐gel mounting medium containing DAPI (FP‐DT094A, Interchim). Images were acquired using a fluorescent microscope (Axioscop Imager.M1, Zeiss, Oberkochen, Germany). Analyses of fiber size, percentage of central myonuclei, and CD68+ cells were manually performed using ImageJ software (NIH, Bethesda, Maryland, USA). To assess muscle fiber cross‐sectional area, a minimum of 300 myofibers per muscle were manually traced. For the analysis of central myonuclei, at least 1000 muscle fibers were evaluated per animal in each condition.

**TABLE 2:** List of antibodies used for Immunohistofluorescence labeling.

Target protein	Dilution	Reference
**CD68**	1/150	*Rabbit anti‐CD68 antibody (ab125212) Abcam*
**FNDC5**	1/150	*Rabbit anti‐FNDC5 antibody (ab174833) Abcam*
**Laminin**	1/300	*Rabbit anti‐laminin antibody (L9393) Sigma Aldrich*
**PAX7**	1/150	*Mouse anti‐PAX7 antibody (ab218472) Abcam*
**Secondary anti‐rabbit Alexa Fluor 488**	1/500	*Goat anti‐rabbit Alexa Fluor 488 (A11008) ThermoFisher*
**Secondary anti‐anti‐rabbit CF Dye 568**	1/500	*Goat anti‐rabbit CF Dye 568 (20102) VWR Biotium*
**Secondary anti‐mouse Alexa Fluor 488**	1/500	*Goat anti‐mouse Alexa Fluor 488 (A11029) ThermoFisher*

### 2.8 Statistical Analysis

All statistical analyses were performed using GraphPad Prism software v9.5.0 (Dotmatics, Boston, MA, USA). Data were tested for normality using the Shapiro–Wilk test and for homogeneity of variances using Fisher's test. For discrete variables and non‐normally distributed data, nonparametric tests (Mann–Whitney *U* test) were applied to determine statistical differences. Parametric tests (Student's *t*‐test) were used for continuous variables and normally distributed data. A two‐way ANOVA was conducted to evaluate the effects of ES on the distribution of fiber cross‐sectional area. When a significant interaction was detected (*p* < 0.05), post hoc multiple comparisons were performed using Šidák's test. For continuous variables and normally distributed data, Pearson's correlation coefficient was employed to assess the strength of relationships between paired data, while Spearman's was used for discrete variables and non‐normally distributed data. Data are presented as means ± standard deviation (SD). For all statistical tests, *p* < 0.05 was considered statistically significant.

## 3 Results

### 3.1 Effect of ES Protocol on Hippocampal BDNF Signaling and Synaptic Protein Expressions

**FIGURE 1:**
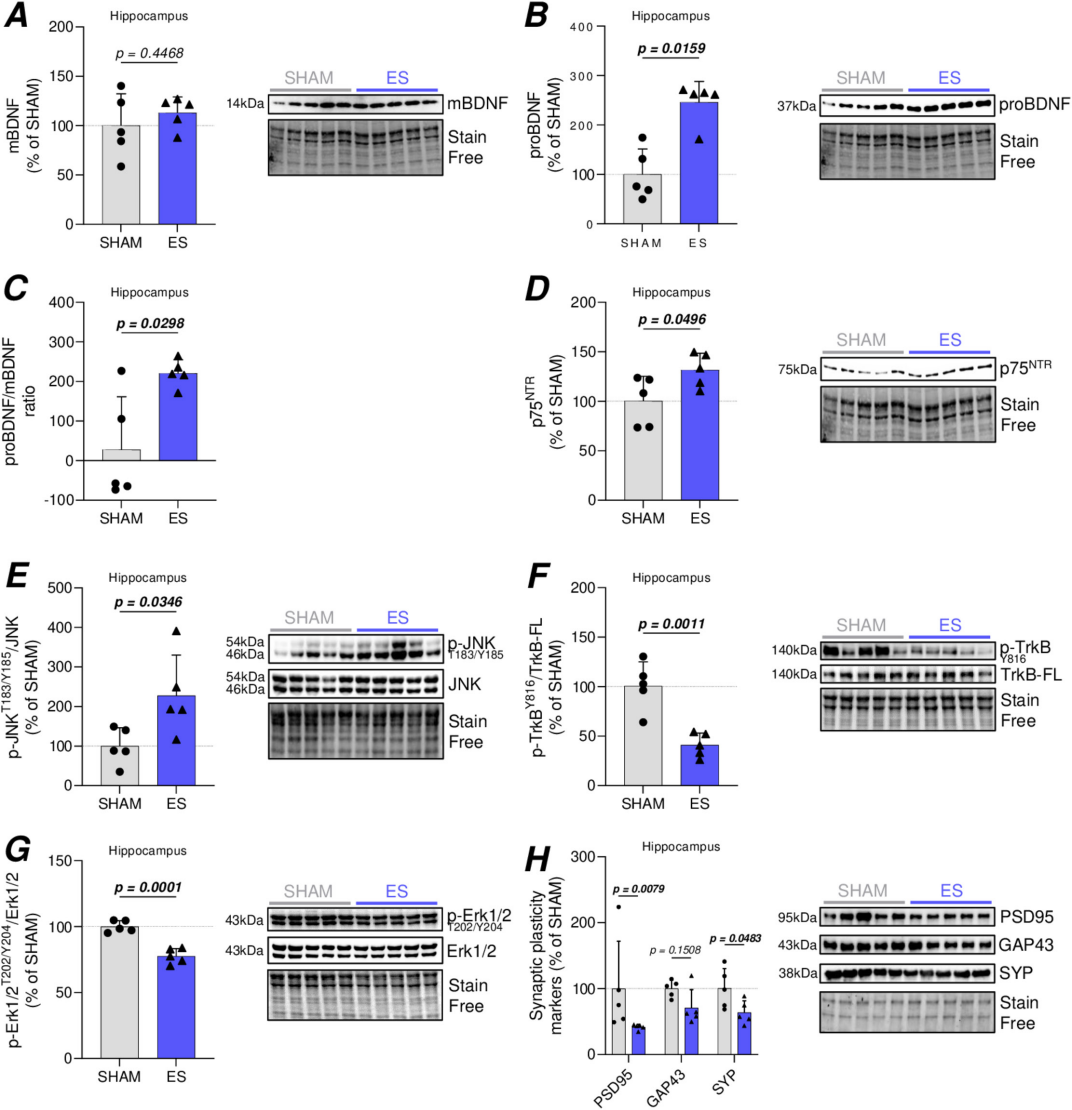
Effect of ES protocol on hippocampal BDNF signaling pathway and synaptic protein expressions. Representative western blot images and quantification of protein levels in SHAM and ES‐treated conditions. (A,B) Hippocampal expression of mBDNF (A) and proBDNF (B). (C) proBDNF/mBDNF ratio based on respective protein expression. (D–H) Expression of p75^NTR^ (D), p‐JNK (T183/Y185) on total JNK (E), p‐TrkB (Y816) on TrkB‐FL (F) and p‐Erk1/2 (T202/Y204) on total Erk1/2 (G), and neuroplasticity markers PSD95, GAP43, and SYP (H). Data are normalized to stain‐free loading controls and expressed as percentage relative to the SHAM group (mean of SHAM set to 100%). Results are represented as mean ± SD of *n* = 5 animals per group. Statistical comparisons were performed using unpaired two‐tailed Student's *t*‐test for panels A, C, D, E, F, and G, and Mann–Whitney *U* test for panels B and H, based on data distribution (normality assessed prior testing). *p* values are indicated above the graphs, in bold, when statistically significant (*p* < 0.05).

To determine whether the ES protocol modulated the BDNF signaling pathway in the hippocampus, we first assessed the levels of proBDNF and mBDNF, along with their respective receptors. As shown in Figure 1, while mBDNF levels remained unchanged (Figure 1A), we observed a significant increase in proBDNF expression in the ES group compared to the SHAM group (Figure 1B), leading to a shift in the pro/mBDNF ratio (Figure 1C). The increase in proBDNF was associated with elevated levels of its receptor p75^NTR^ (Figure 1D), which can activate pro‐apoptotic JNK signaling upon binding (Chao 2003). Consistent with this, we detected an increase in JNK activation in the hippocampus of the ES group compared to the SHAM group, with no change in total JNK levels (Figure 1E). However, despite the increase in JNK activation, no significant alterations were found in the expression of cytochrome c or caspase‐3 activation (Figure S1B,C).

Given the observed alterations in proBDNF and p75^NTR^ levels, we next investigated the impact of our protocol on TrkB signaling—a cellular pathway mediating the beneficial effects of mBDNF. ES significantly reduced TrkB phosphorylation without affecting the total expression levels of the full‐length (FL) form (Figure 1F). Typically, TrkB activation by mBDNF leads to the downstream activation of Akt and Erk1/2—key molecules regulating neuron survival and synaptic function (Y. Wang et al. 2024). Consistent with the reduced TrkB phosphorylation, we found that ES diminished Erk1/2 activation in the hippocampus (Figure 1G), while Akt activation remained unchanged (Figure S1D).

Considering the observed changes in BDNF‐related signaling pathways, we analyzed several markers of synaptic plasticity, including growth‐associated protein 43 (GAP43), postsynaptic density protein 95 (PSD95), and synaptophysin (SYP), which are widely used to assess synaptic activity and synaptogenesis and are typically upregulated in response to traditional exercise (Guo et al. 2023). Our results indicate a significant reduction in hippocampal PSD95 and SYP expressions, along with a downward trend in GAP43 levels in the ES group compared to SHAM animals (Figure 1H), suggesting that synaptic plasticity may be impaired following ES‐induced muscle injury.

### 3.2 Effect of ES Protocol on Molecular Mechanisms Underlying Hippocampal BDNF Expression

**FIGURE 2:**
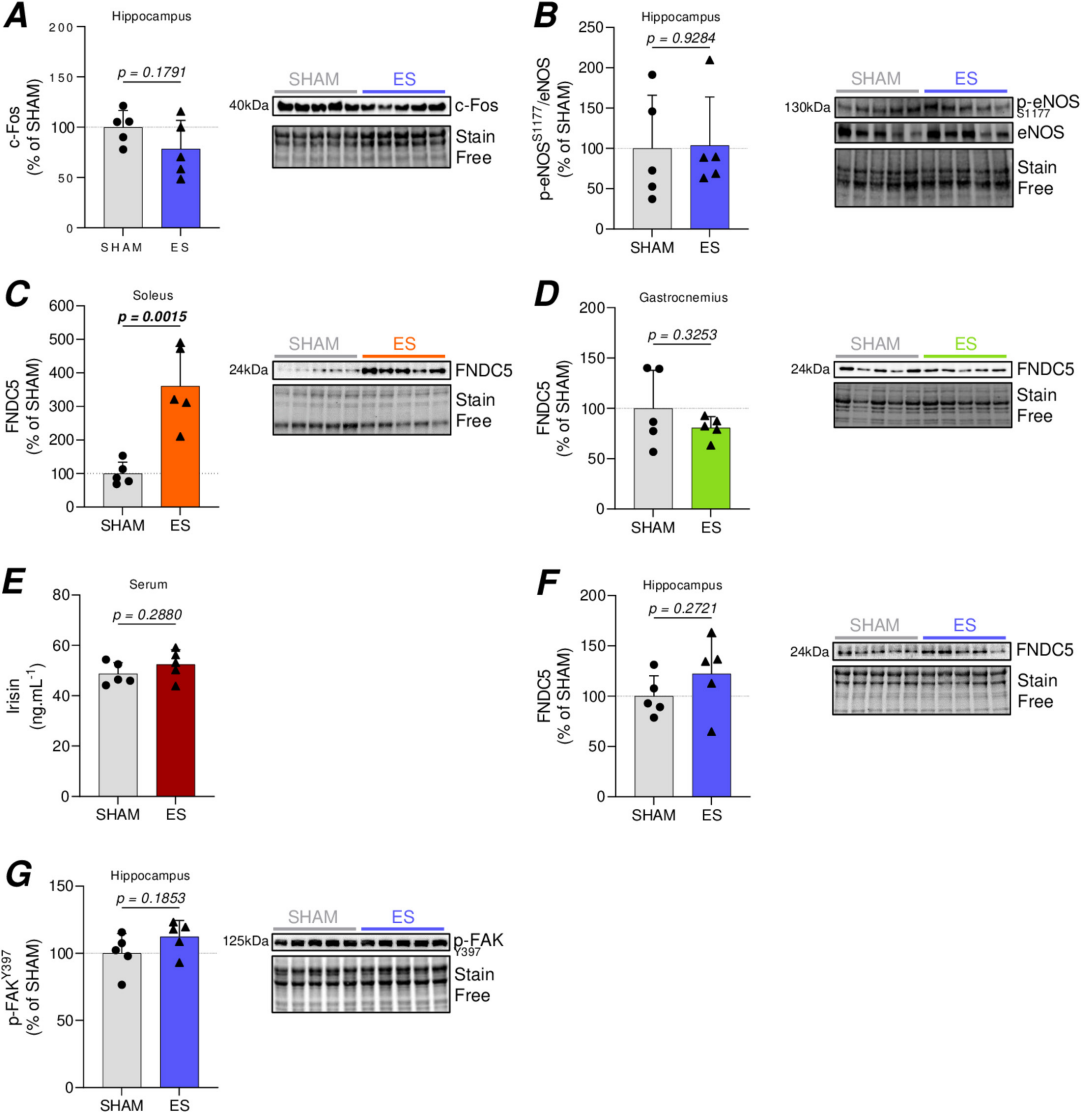
Effect of ES protocol on molecular mechanisms underlying hippocampal BDNF expression. Representative western blot images and quantification of protein levels in SHAM and ES‐treated conditions. (A–D) Hippocampal expression of c‐Fos (A) and p‐eNOS (S1177) (B). FNDC5 expression in the soleus (C) and gastrocnemius (D). (E) Circulating levels of irisin. (F‐G) Hippocampal expression of FNDC5 (F) and p‐FAK (Y397) (G). Western blotting data are normalized to stain‐free loading controls and expressed as percentage relative to the SHAM group (mean of SHAM set to 100%). Results are represented as mean ± SD of *n* = 5 animals per group. Statistical analysis was performed using unpaired two‐tailed Student's *t‐*test for each comparison (A–G). *p* values are indicated above the graphs, in bold, when statistically significant (*p* < 0.05).

To understand the mechanisms underlying the maladaptive changes in hippocampal BDNF signaling, we investigated the effects of ES on neuronal activity and hemodynamic shear stress in the hippocampus, both known regulators of BDNF expression (Cefis et al. 2022; Marie et al. 2018). We found no significant difference between ES and SHAM groups in the expression of c‐Fos (Figure 2A)—a marker of neuronal activation (Cruz‐Mendoza et al. 2022)—and p‐eNOS^S1177^ (Figure 2B)—a marker activated in response to exercise‐induced shear stress (Cefis et al. 2022; Tryfonos et al. 2023). These data suggest that the observed changes in hippocampal BDNF signaling are not a consequence of altered neuronal activity or cerebral blood flow induced by the ES protocol. To determine whether the observed changes in hippocampal BDNF signaling could be mediated by the FNDC5/Irisin pathway, we assessed FNDC5 expression in both the soleus (SOL) and gastrocnemius (GAS) muscles, as well as circulating irisin serum levels. Our data revealed that FNDC5 expression was significantly increased in the SOL of ES animals (Figure 2C), while no change was observed in the GAS muscle (Figure 2D). Similarly, circulating irisin levels were unchanged 24 h after the final stimulation session (Figure 2E). As irisin can cross the BBB and bind to the αVβ5 integrin receptor, thereby triggering focal adhesion kinase (FAK)‐dependent signaling cascades (A et al. 2023), we also measured the hippocampal protein expression of FNDC5 and p‐FAK^Y397^ and observed no significant differences between SHAM and ES animals (Figure 2F,G), suggesting that irisin signaling in the hippocampus was not altered by this protocol.

### 3.3 Histological Assessment of ES‐Induced Muscle Alterations

**FIGURE 3:**
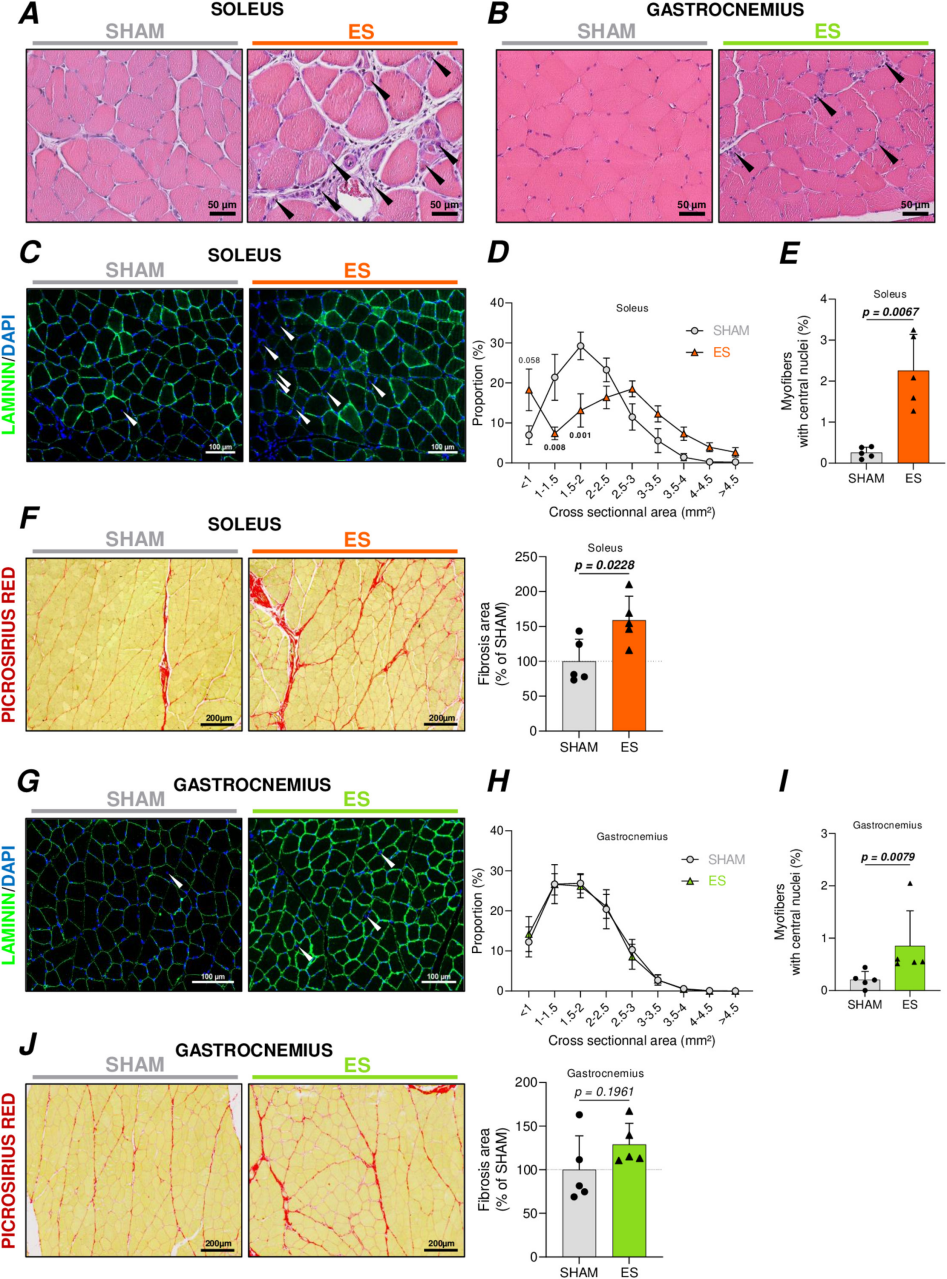
Histological assessment of ES‐induced muscle alterations. (A,B) Representative hematoxylin/eosin‐stained immunohistochemistry images of soleus (A) and gastrocnemius (B) in SHAM and ES‐treated rats (black arrows show very small muscle fibers or inflammatory cells infiltration). (C–E) Soleus cross‐sectional laminin and DAPI immunolabeling (white arrows point to fibers with centrally located nuclei) (C) and analyses of cross‐sectional area (D) and central myonuclei (E). (F) Representative picrosirius red‐stained immunohistochemistry images of soleus and analysis of fibrosis extent. (G–I) Gastrocnemius cross‐sectional laminin and DAPI immunolabeling (white arrows show fibers with centrally located nuclei) (G) and analyses of cross‐sectional area (H) and central myonuclei (I). (J) Representative picrosirius red‐stained immunohistochemistry images of gastrocnemius and analysis of fibrosis extent. Panels E and I are represented as the proportion of fibers with central nuclei relative to the total number of fibers analyzed, and panels F and J are expressed as a percentage relative to the SHAM group (Mean SHAM = 100%). Statistical comparisons were performed using unpaired two‐tailed Student's *t*‐test for panels E, F, and J; Mann–Whitney *U* test for panel I; and two‐way ANOVA followed by Sidak's post hoc test for panels D and H, based on data distribution (normality assessed prior testing). *p* values are indicated above the graphs, in bold, when statistically significant (*p* < 0.05).

Since ES protocol is unlikely to influence irisin‐dependent hippocampal signaling, we next investigated whether our protocol induced any local changes within skeletal muscle tissue by performing histological analyses on both SOL and GAS muscles. Our analyses revealed notable alterations in ES‐exposed animals (Figure 3A,B). Specifically, in the SOL muscle, we observed numerous fragmented muscle fibers, damaged sarcoplasm, and inflammatory cell infiltration compared to SHAM animals (Figure 3A). These features were less pronounced in the GAS muscle (Figure 3B). To further characterize muscle integrity, we analyzed the fiber cross‐sectional area (CSA), the proportion of central myonuclei, and the extent of fibrosis in both the SOL and GAS muscles. In the SOL, our data revealed that our protocol induced a switch in the fiber CSA distribution with a significant increase in the number of small CSA (< 1 mm^2^) fibers and a concomitant decrease of intermediate CSA fibers (from 1 to 2.5 mm^2^), indicative of muscle fiber regeneration following the ES protocol (Figure 3C,D). Consistently, our data also revealed that the proportion of fibers with central myonuclei was significantly increased in the SOL muscle of ES animals as compared to SHAM animals (Figure 3C–E). Finally, picrosirius red staining to detect collagen revealed an increase in collagen deposition and thus fibrosis in the SOL muscle of ES animals (Figure 3F). In the GAS, structural integrity was less impacted by the ES protocol compared to the SOL. The fiber CSA distribution did not differ significantly from SHAM animals, the increase in centralized nuclei was less pronounced, and picrosirius red staining showed no significant fibrosis relative to SHAM controls (Figure 3G–J). Collectively, these results indicate that the ES protocol used in our study alters the SOL muscle and to a lesser extent the GAS muscle as evidenced by the significantly greater proportion of central myonuclei, collagen deposition, and CD68+ cells observed in SOL muscle compared to GAS muscle (Figure S2A–C).

### 3.4 Involvement of FNDC5 in ES‐Induced Muscle Regeneration and Repair

**FIGURE 4:**
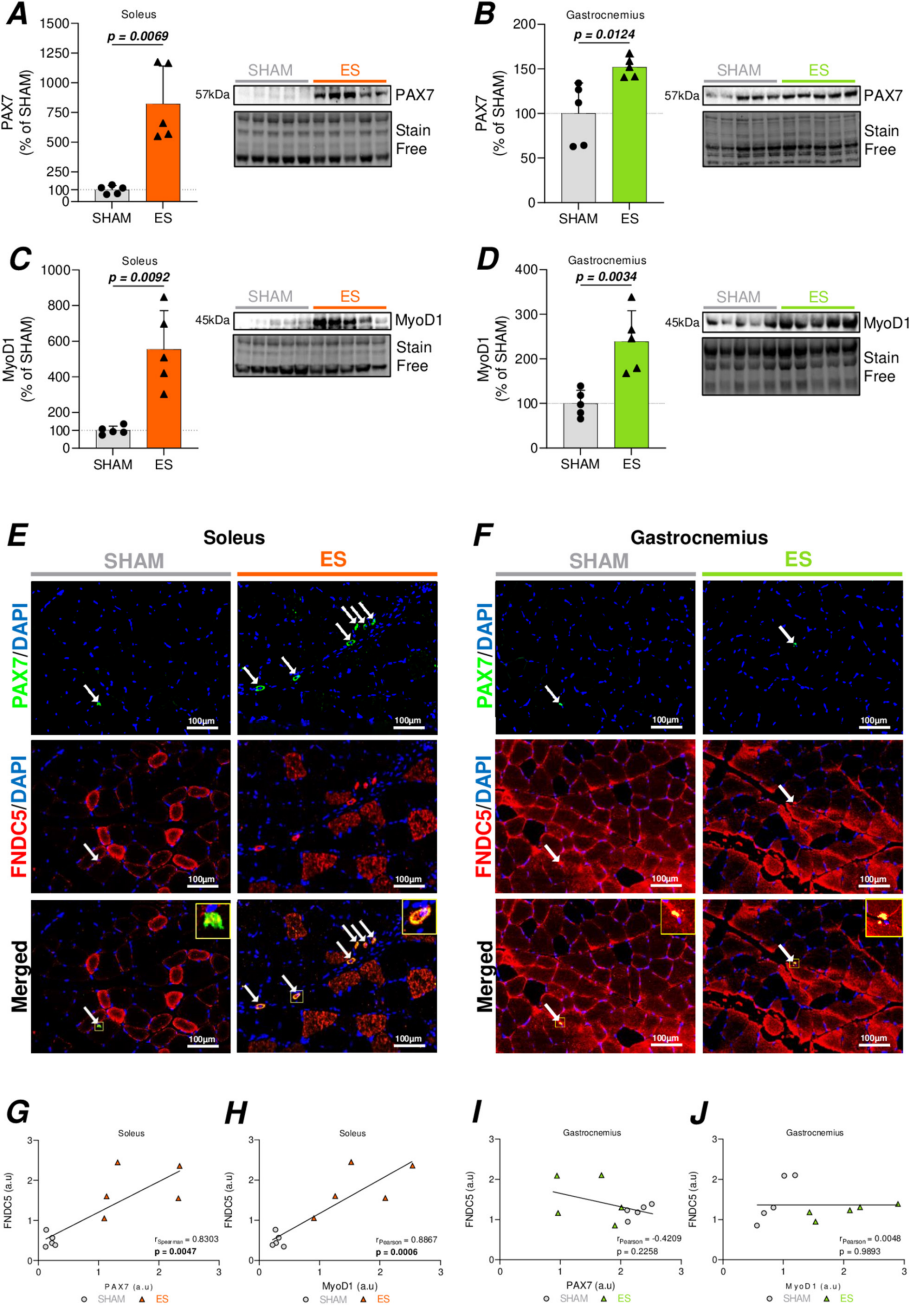
Involvement of FNDC5 in ES‐induced muscle regeneration and repair. (A–D) Representative western blot images and quantification of protein levels in SHAM and ES‐treated conditions of PAX7 (A, B) and MyoD1 (C, D) in the soleus (A–C) and the gastrocnemius (B–D). (E‐F) Representative images of immunohistofluorescence labeling for PAX7/FNDC5 in soleus (E) and gastrocnemius (F). (G–J) Correlations between FNDC5 and muscle satellite cells specific proteins in soleus (G, H) and gastrocnemius (I, J) muscles. Spearman or Pearson correlations between expression of FNDC5/PAX7 (G) and FNDC5/MyoD1 (H) in soleus, and FNDC5/PAX7 (I) and FNDC5/MyoD1 (J) in gastrocnemius. For Western blotting analyses, data are normalized to stain‐free loading controls and expressed as percentage relative to the SHAM group (mean of SHAM set to 100%). Results are represented as mean ± SD of *n* = 5 animals per group. Statistical analysis was performed using unpaired two‐tailed Student's *t*‐test for each comparison (A–D). *p* values are indicated on the graphs, in bold, when statistically significant (*p* < 0.05).

Following our findings that both the SOL and, to a lesser extent, the GAS muscles displayed key signs of muscle regeneration and repair, we next assessed muscle satellite cell (MuSC) density and differentiation through the protein expression of PAX7 and MyoD1, respectively. Our data revealed that ES significantly increased PAX7 (Figure 4A,B) and MyoD1 (Figure 4C,D) expression in both muscles, with a stronger relative increase observed in the SOL (+722% for PAX7 and +454% for MyoD1) as compared to the GAS muscle (+52% for PAX7 and +139% for MyoD1).

Additionally, given that irisin injection has been shown to promote MuSC activation upon muscle injury (Reza et al. 2017), we performed immunostaining analysis and found that FNDC5 co‐localized with PAX7 staining in both muscle groups, with a greater number of co‐labeled MuSC in the SOL (Figure 4E,F). Positive correlations between FNDC5 and PAX7/MyoD1 expression levels were observed in the SOL muscle (Figure 4G) but not in the GAS muscle (Figure 4H). Taken altogether, these results suggest that part of FNDC5 expression could also originate from MuSC.

### 3.5 ES‐Induced Muscle Pro‐Inflammatory Status Contributes to Altered Hippocampal BDNF Signaling

**FIGURE 5:**
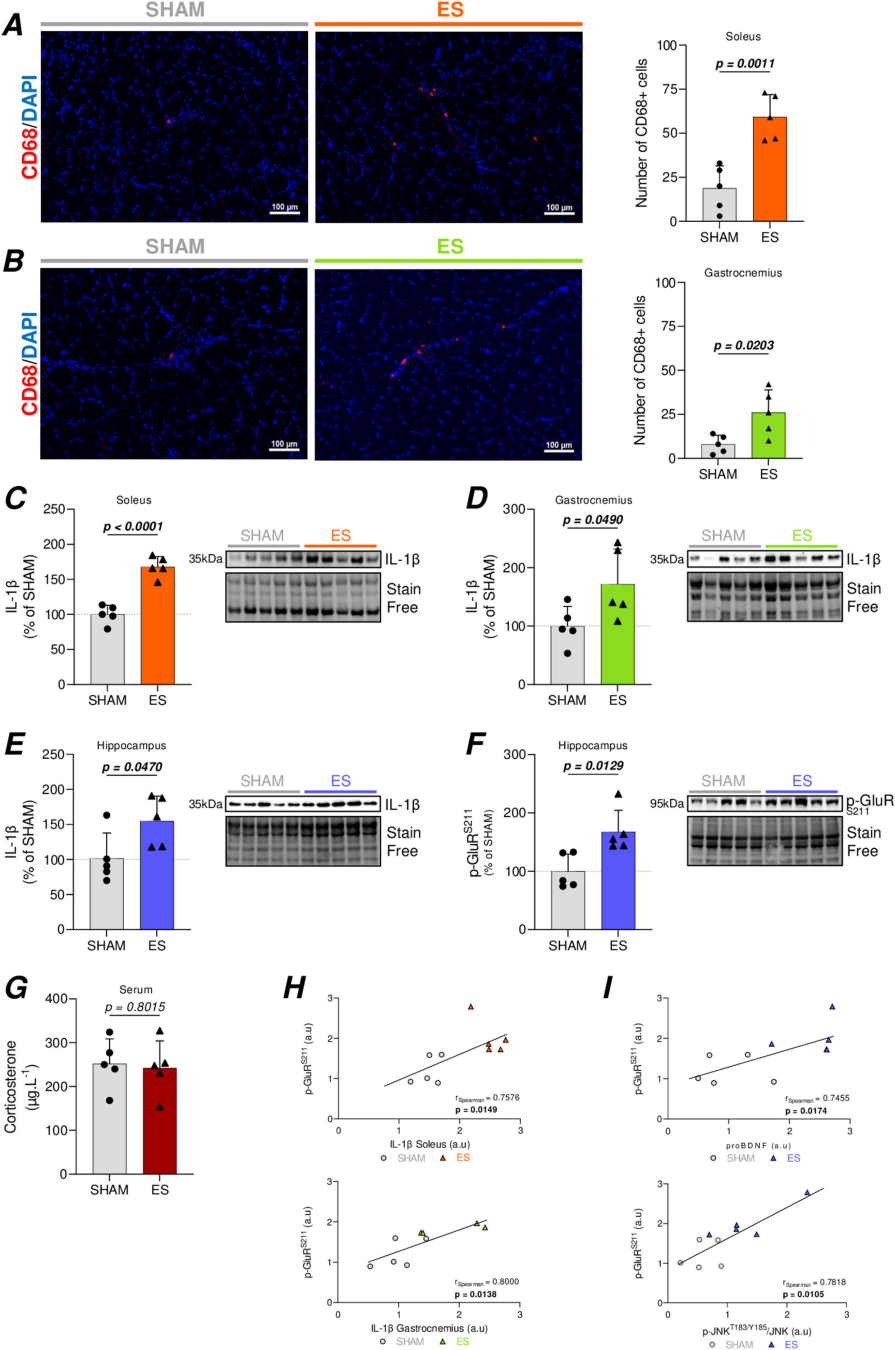
ES‐induced muscle pro‐inflammatory status, contributes to altered hippocampal BDNF signaling. (A,B) Representative images of immunohistofluorescence labeling and quantification for CD68 in soleus (A) and gastrocnemius muscles (B). CD68+ cell counts represent the total number of positive cells identified across full muscle cross‐section for each animal. (C–F) Representative western blot images and quantification of protein levels in SHAM and ES‐treated conditions of IL‐1β in the soleus (C), gastrocnemius (D), hippocampus (E), and p‐GluR (S211) hippocampal expression (F). Circulating levels of corticosterone (G). Spearman correlation between hippocampal expression of p‐GluR (S211) and IL‐1β expression in the soleus (up) or gastrocnemius (down) (H). Spearman correlation between hippocampal expression of p‐GluR (S211) and proBDNF (up) or p‐JNK (T183/Y185) on total JNK ratio (down) for sham animals (circle) and ES‐treated rats (triangle) (I). For Western blotting analyses, data are normalized to stain‐free loading controls and expressed as percentage relative to the SHAM group (mean of SHAM set to 100%). Results are represented as mean ± SD of *n* = 5 animals per group. All statistical comparisons were performed using unpaired two‐tailed Student's *t*‐test for each comparison. *p* values are indicated on the graphs, in bold, when statistically significant (*p* < 0.05).

To assess the pro‐inflammatory status of both muscles, we quantified CD68+ cells as an index of inflammatory cell infiltration and measured IL‐1β protein expression—a pro‐inflammatory cytokine known to interfere with BDNF‐induced neuroplasticity (Carlos et al. 2017; Tong et al. 2008). Our data showed that the number of CD68+ cells was significantly increased by ES in both muscles (Figure 5A,B). Despite no difference in circulating levels (Figure S1E), we also observed an increase in muscular IL‐1β expression upon ES (Figure 5C,D) along with an increase in hippocampal expression of this cytokine (Figure 5E). To determine whether these enhancements were associated with neuroinflammatory mechanisms within the brain, we examined the hippocampal expression of GFAP and Iba1, which serve as markers for astrocytic and microglial reactivity, respectively. No significant differences were observed between the SHAM and ES groups in the expression of either GFAP or Iba1, suggesting that the ES protocol is not associated with a reactive gliosis response (Figure S1F,G).

Given the evidence of muscle inflammation and its impact on brain BDNF signaling, we examined the activation of hippocampal glucocorticoid receptors (GluR)—a key component of the stress response (Srinivasan et al. 2013). Our data revealed that the ES protocol induced an activation of the GluR (Figure 5F), whereas corticosterone measured at the same time point (24 h after the last stimulation session) was not different between the two groups of animals (Figure 5G). Further correlation analyses revealed a positive association between muscle IL‐1β expression and hippocampal glucocorticoid receptor activation (Figure 5H), while within the hippocampus, glucocorticoid receptor activation was also positively associated with changes in the expression of proBDNF and p‐JNK (Figure 5I). In conclusion, our results suggest a potential connection between ES‐induced muscle damage, GluR activation, and alterations in BDNF signaling pathways.

## 4 Discussion

This study investigated whether chronic ES of hindlimb muscles could mimic the beneficial effects of exercise on hippocampal BDNF signaling and neuroplasticity, with a particular focus on the FNDC5/Irisin pathway.

Contrary to our hypothesis, ES caused significant muscle injury, a proinflammatory response, and alterations in hippocampal BDNF signaling, suggesting maladaptive muscle‐brain crosstalk. Chronic ES led to an increase in proBDNF expression without altering mBDNF levels. This elevated proBDNF/mBDNF ratio was associated with increased p75^NTR^ expression and JNK activation, indicating enhanced proBDNF signaling (Luo et al. 2019; Teng et al. 2005). Consistently, the ES protocol reduced TrkB activation without changing TrkB‐FL expression and decreased downstream Erk1/2 activation, which aligns with data on chronically stressed rats (Bai et al. 2016). Furthermore, key neuroplasticity markers (i.e., GAP43, PSD95, and SYP) were downregulated in ES animals, in line with the effects of chronic stress on BDNF signaling and mood regulation (Liu et al. 2020). While proBDNF signaling can have pro‐apoptotic effects, we found no significant change in hippocampal cytochrome c and caspase‐3 expression and cleavage (Troy et al. 2002). Despite JNK activation, this finding suggests that ES‐induced muscle damage does not trigger downstream apoptotic pathways in the brain. Instead, JNK activation may reflect early neuronal dysfunction, as observed in Alzheimer's disease models (Priori et al. 2023).

To explore the molecular mechanisms underlying alterations in hippocampal BDNF signaling, we examined the effects of ES on neuronal activity and hemodynamic responses. The lack of difference in c‐Fos or p‐eNOS^S1177^ expressions between ES and control groups suggests that the disruption in BDNF signaling was not driven by major changes in neuronal activation or hemodynamic responses. We then explored the potential involvement of the humoral pathway, which has recently been implicated in the mechanism underlying exercise‐induced BDNF overexpression, particularly through the irisin muscle‐brain signaling axis, in mediating these effects (Ieraci et al. 2016; Wrann et al. 2013). While ES robustly increases FNDC5 expression in the SOL (but not in the GAS), neither circulating irisin nor hippocampal FNDC5 expression increased. This contrasts with findings from classical exercise models reporting increased circulating irisin and activation of the hippocampal FNDC5 pathway (Huh et al. 2012; Wrann et al. 2013). Additionally, we observed no changes in FAK activation, suggesting that this pathway, downstream of the αV/β5 integrin receptors, remains inactive, although this pathway has been proposed to facilitate irisin‐mediated communication within the brain (Islam et al. 2021; Y. Wang, Tian, et al. 2022). The FNDC5 increase in the SOL—a slow‐twitch muscle—was also unexpected, as previous studies suggest that FNDC5/Irisin overexpression originates predominantly from fast‐twitch fibers (Dupuis et al. 2024; Leger et al. 2024). Given that irisin promotes muscular stem cell proliferation and tissue regeneration (Reza et al. 2017), we examined the effects of ES on muscle integrity. Contrary to earlier findings suggesting minimal myofiber cell death from NMES protocols (Toth et al. 2020), our data indicate that the ES protocol substantially compromised muscle structural integrity, with the SOL exhibiting more pronounced alterations than the GAS, consistent with its susceptibility to exercise‐induced damages (Lloyd et al. 2023). In this respect, in view of its severity, our model appears more comparable to chemical or physical models of muscle damage than to conventional NMES models, although the extent of damage (e.g., perturbations in fiber cross‐sectional area distribution, increased centralized myonuclei, and fibrosis) aligns with human studies reporting significant rhabdomyolysis following whole‐body NMES (Kästner et al. 2015). Thus, elevated FNDC5 expression may reflect the activation of intrinsic repair mechanisms, rather than activation of a systemic humoral pathway. This interpretation is further supported by increased PAX7 and MyoD1 expressions—markers of satellite cell proliferation and differentiation—and the localization of FNDC5 expression with PAX7‐positive cells, suggesting a potential autocrine/paracrine role for FNDC5/Irisin in muscle regeneration. Although correlation does not imply causation, the positive association between FNDC5 and PAX7 or MyoD1 expression levels in the SOL further supports the involvement of FNDC5/Irisin in muscle repair. Taken together, these results align with the known function of this protein family since FNDC4 (Li et al. 2020; Z. Wang et al. 2020) and FNDC1 (Zhang et al. 2025) have been reported as key regulators of myogenic differentiation and skeletal muscle regeneration. However, FNDC5 expression might also regulate the inflammatory milieu by modulating cytokine production and influencing macrophage polarization, as demonstrated in models of inflammation‐mediated metabolic syndrome (Slate‐Romano et al. 2022).

To better understand the disturbances between muscle damage and altered hippocampal signaling, we examined the muscular inflammatory state. The ES protocol increased CD68+ inflammatory cell labeling and upregulated the pro‐inflammatory cytokine IL‐1β in muscle tissue. While IL‐1β levels were also elevated in the hippocampus, we found no detectable changes in circulating IL‐1β. Given the well‐established link between peripheral inflammation and neuroinflammation (Sun et al. 2022), we further investigated the gliosis response. Unlike findings from models of systemic intestinal inflammation (Zonis et al. 2015) or LPS‐induced neuroinflammation (Fu et al. 2014), astrocytic and microglial marker levels remained unchanged. Taken together, these results suggest that the ES protocol did not induce widespread neuroinflammation but only subtle changes in BDNF signaling. Like chronic stress models (Agarwal et al. 2021; Xiang et al. 2015), ES led to glucocorticoid receptor activation, possibly through hypothalamic–pituitary–adrenal (HPA) axis activation (Lou et al. 2018). Interestingly, muscular IL‐1β expression was positively associated with glucocorticoid receptor activation, which in turn correlated with proBDNF levels and signaling, consistent with studies demonstrating that stress disrupts hippocampal synaptic plasticity by altering the proBDNF/mBDNF balance (Bai et al. 2016; Yeh et al. 2012). Collectively, our results point to a muscle‐derived, inflammation‐linked signal that initiates a maladaptive muscle–brain dialogue, impairing hippocampal BDNF signaling without eliciting extensive neuroinflammatory activation.

Several limitations should be considered when interpreting our findings. First, our study employed a single ES protocol, which turned out to be quite severe and is finally more closely related to chemical or physical muscle damage models, and we cannot exclude that different parameters (e.g., frequency, intensity, and duration) may yield different outcomes. Second, we did not evaluate the temporal profile (kinetics) of systemic irisin, IL‐1β, and corticosterone expression and thus cannot exclude transient elevation of these factors (e.g., immediately after stimulation). Third, we did not assess cognitive function directly, so we cannot conclude that the observed changes in hippocampal BDNF signaling translate into cognitive impairments. However, the significant muscle damage observed in ES animals, combined with our strict adherence to ethical standards, precluded the inclusion of additional animals. Finally, another limitation of our methodology is that stimulation intensity was adjusted based on visual estimation of joint movement and muscle response rather than direct torque measurements.

## 5 Conclusion

In conclusion, our study demonstrates that chronic ES of rat hindlimb muscles, intended to mimic exercise‐induced central benefits, induces muscle injury that negatively affects BDNF‐dependent neuroplasticity, likely by triggering a maladaptive muscle‐brain crosstalk. The increased FNDC5 within damaged muscle appears to reflect local repair mechanisms rather than a beneficial systemic signal. Although our model severity excludes its application to humans, our data underscore the importance of optimizing muscle stimulation protocols for application in populations unable to engage in conventional physical activity. Further research should identify ES parameters that foster beneficial muscle‐brain communication without causing muscle damage, particularly in individuals with pre‐existing muscle weakness.

## Author Contributions

**Julien Wirtz:** data curation, formal analysis, investigation, methodology, project administration, resources, software, validation, visualization, writing – original draft, writing – review and editing. **Rémi Chaney:** data curation, formal analysis, funding acquisition, investigation, methodology, project administration, resources, supervision, validation, visualization, writing – original draft, writing – review and editing. **Marina Cefis:** data curation, formal analysis, investigation, methodology, project administration, resources, supervision, validation, writing – original draft, writing – review and editing. **Alexandre Méloux:** investigation, methodology, resources, writing – review and editing. **Stéphanie Lemaire:** formal analysis, investigation, resources, software. **Aurore Quirié:** investigation, methodology, resources. **Julien Delezie:** writing – review and editing. **Gilles Gouspillou:** writing – review and editing. **Anne Prigent‐Tessier:** conceptualization, funding acquisition, methodology, project administration, resources, supervision, validation, writing – original draft, writing – review and editing. **Philippe Garnier:** conceptualization, funding acquisition, methodology, project administration, resources, supervision, validation, writing – original draft, writing – review and editing.

## Ethics Statement

The experiments were conducted in accordance with guidelines set forth by the French Department of Agriculture (licence 21‐CAE‐102) and approved by the local ethics committee (Ethics Committee of Animal Experimentation, Dijon, registration number 105).

## Conflicts of Interest

The authors declare no conflicts of interest.

## Peer Review

The peer review history for this article is available at https://www.webofscience.com/api/gateway/wos/peer‐review/10.1111/ejn.70235.

## Supporting information


**Figure S1:** (A) Whole body, soleus, and medial gastrocnemius masses in SHAM animals and ES‐treated rats. (B–D) Relative protein expression of hippocampal cytochrome c (B), cleaved‐caspase‐3 on total caspase‐3 ratio (C), and p‐Akt (S473) (D). (E) Circulating levels of IL‐1β. (F, G) Relative protein expression of hippocampal GFAP (F) and Iba1 (G). For Western blotting analyses, data are normalized to stain‐free loading controls and expressed as percentage relative to the SHAM group (mean of SHAM set to 100%). Results are represented as mean ± SD of *n* = 5 animals per group. All statistical comparisons were performed using unpaired two‐tailed Student's *t*‐test for each comparison. *p* values are indicated on the graphs, in bold, when statistically significant (*p* < 0.05).

**Figure S2:** (A) Comparison between soleus and gastrocnemius muscles for central myonuclei, represented as the proportion of fibers with central nuclei relative to the total number of fibers counted. (B) Comparison between soleus and gastrocnemius muscles in fibrosis extent, represented as the ratio of the fibrosis area on the total muscle cross‐section area. (C) Comparison between soleus and gastrocnemius muscles in CD68+ cell counts, represented as the total number of positive cells identified across full muscle cross‐section for each animal. Results are represented as mean ± SD of *n* = 5 animals per group. All statistical comparisons were performed using unpaired two‐tailed Student's *t*‐test for each comparison. *p* values are indicated on the graphs, in bold, when statistically significant (*p* < 0.05).

**Data S1:** Supporting information.

## Data Availability

The raw data supporting the conclusions of this article will be made available by the authors, without undue reservation.
