# Supplementary figures and images for: Electrical Stimulation‐Induced Muscle Damage Alters Hippocampal BDNF Signaling
Source: Eur J Neurosci. 2025 Sep 17;62(6):e70235. doi: 10.1111/ejn.70235 (PMC12442748; doi:10.1111/ejn.70235)

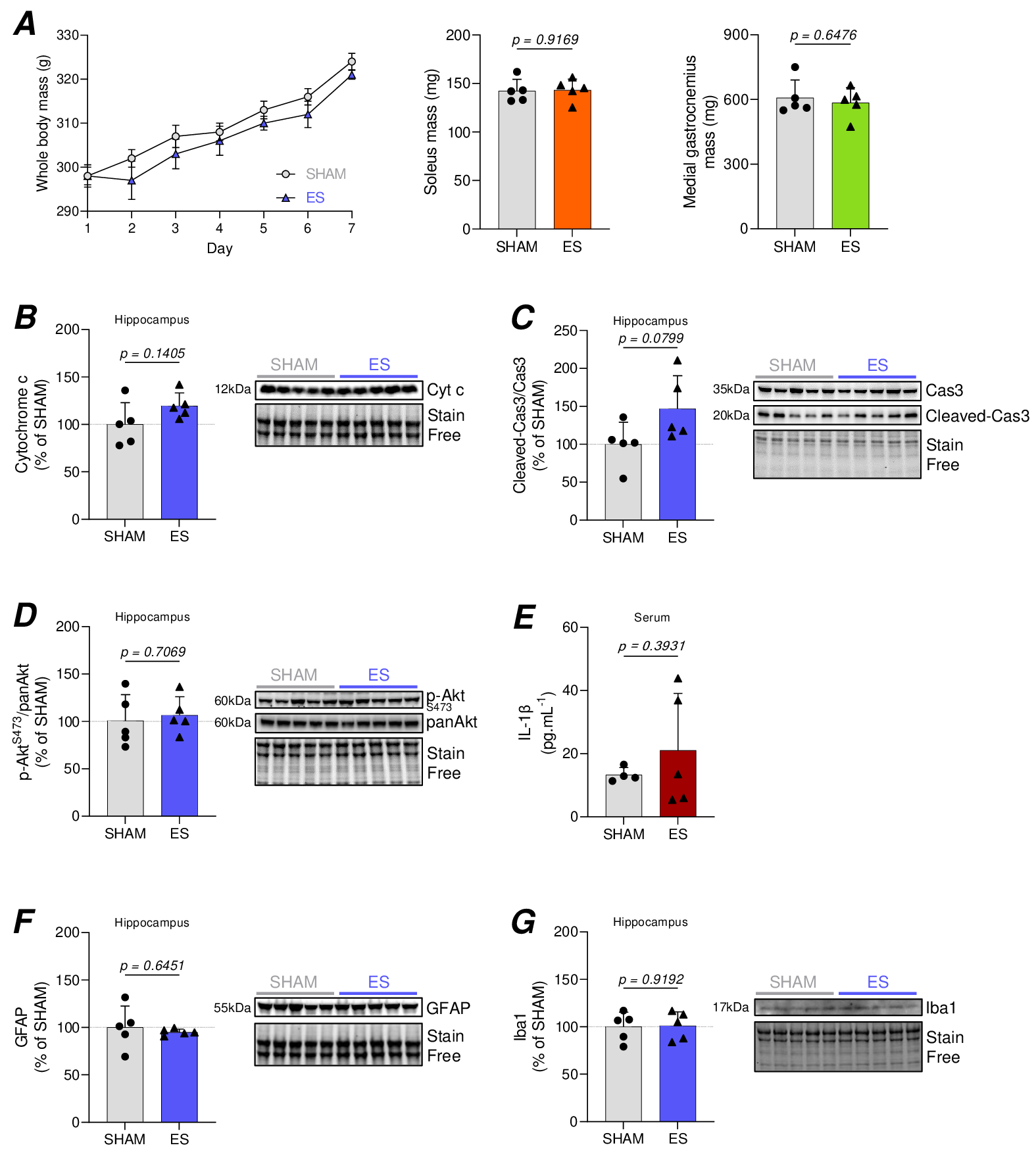

Supplement: Supplementary file 1 — Figure S1: (A) Whole body, soleus, and medial gastrocnemius masses in SHAM animals and ES‐treated rats. (B–D) Relative protein expression of hippocampal cytochrome c (B), cleaved‐caspase‐3 on total caspase‐3 ratio (C), and p‐Akt (S473) (D). (E) Circulating levels of IL‐1β. (F, G) Relative protein expression of hippocampal GFAP (F) and Iba1 (G). For Western blotting analyses, data are normalized to stain‐free loading controls and expressed as percentage relative to the SHAM group (mean of SHAM set to 100%). Results are represented as mean ± SD of n = 5 animals per group. All statistical comparisons were performed using unpaired two‐tailed Student's t‐test for each comparison. p values are indicated on the graphs, in bold, when statistically significant (p < 0.05). [file EJN-62-0-s003.tiff]

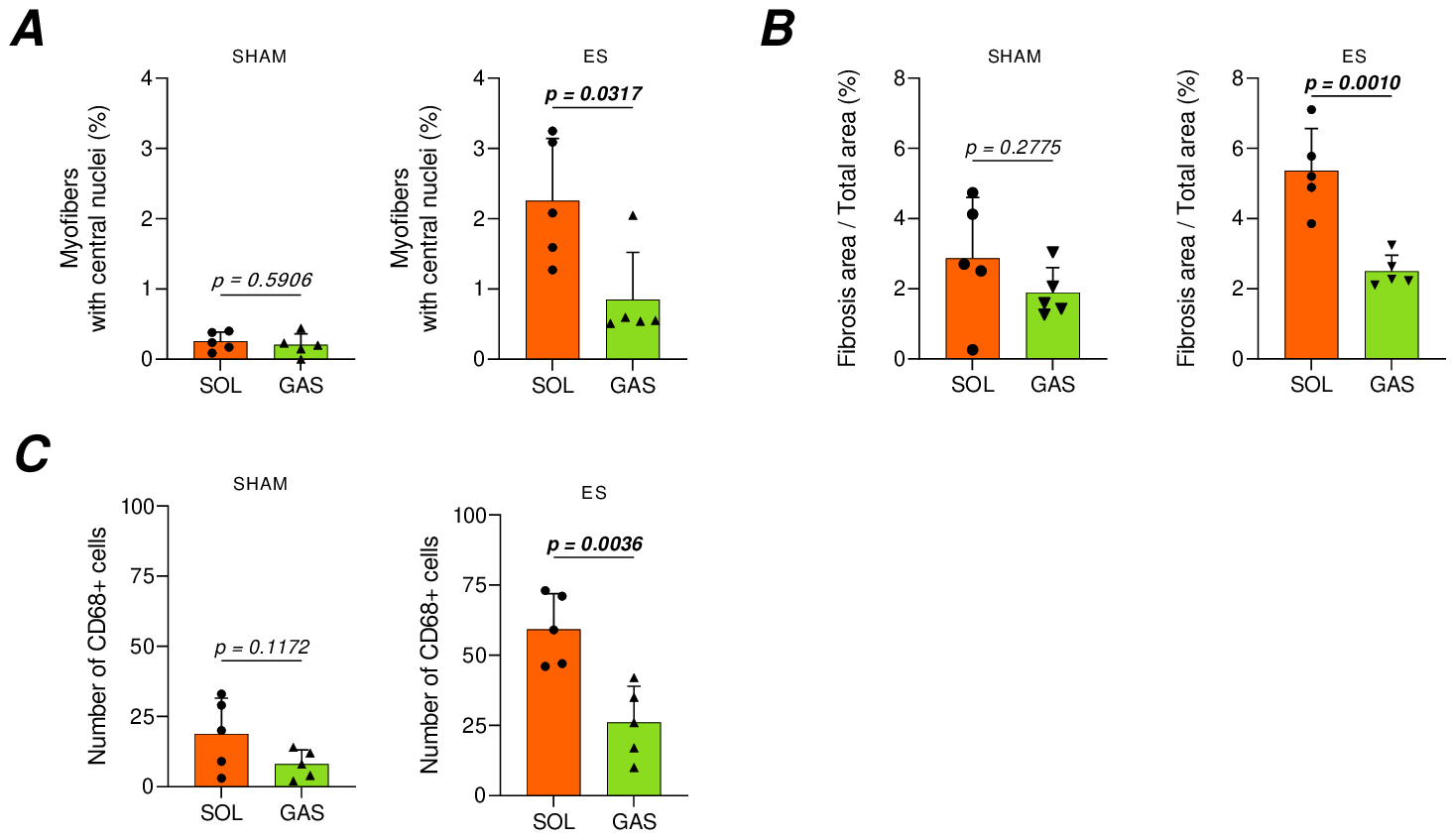

Supplement: Supplementary file 2 — Figure S2: (A) Comparison between soleus and gastrocnemius muscles for central myonuclei, represented as the proportion of fibers with central nuclei relative to the total number of fibers counted. (B) Comparison between soleus and gastrocnemius muscles in fibrosis extent, represented as the ratio of the fibrosis area on the total muscle cross‐section area. (C) Comparison between soleus and gastrocnemius muscles in CD68+ cell counts, represented as the total number of positive cells identified across full muscle cross‐section for each animal. Results are represented as mean ± SD of n = 5 animals per group. All statistical comparisons were performed using unpaired two‐tailed Student's t‐test for each comparison. p values are indicated on the graphs, in bold, when statistically significant (p < 0.05). [file EJN-62-0-s002.tiff]
