# Supplementary material for: Electrical Stimulation‐Induced Muscle Damage Alters Hippocampal BDNF Signaling
Source: Eur J Neurosci. 2025 Sep 17;62(6):e70235. doi: 10.1111/ejn.70235 (PMC12442748; doi:10.1111/ejn.70235)
Supplement: Supplementary file 3 — Data S1: Supporting information. [file EJN-62-0-s001.pdf]

# Full unedited blots for Figure 1

## 1.A-B

proBDNF & mBDNF

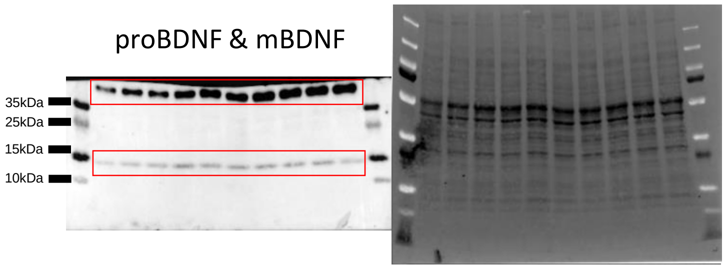


## 1.D

$p75^{NTR}$

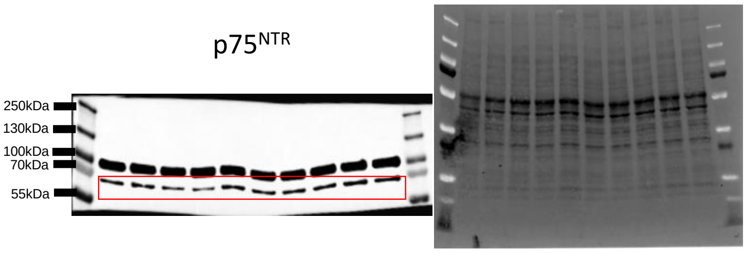


## 1.E

$P\text{-}JNK^{T183/Y185}$

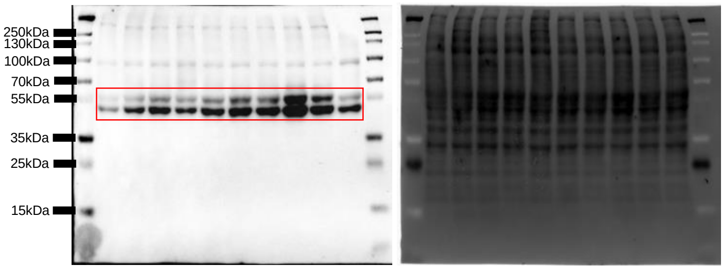


## 1.E

JNK

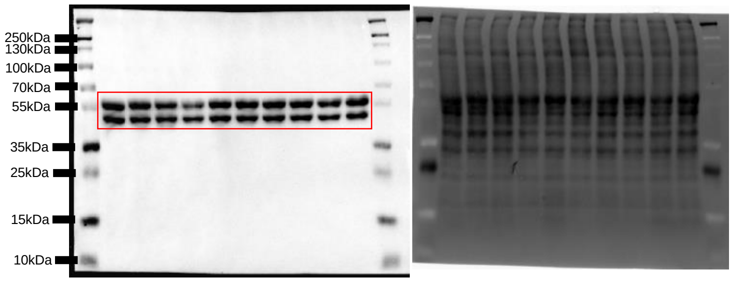


## 1.F

$P\text{-}TrkB^{Y816}$

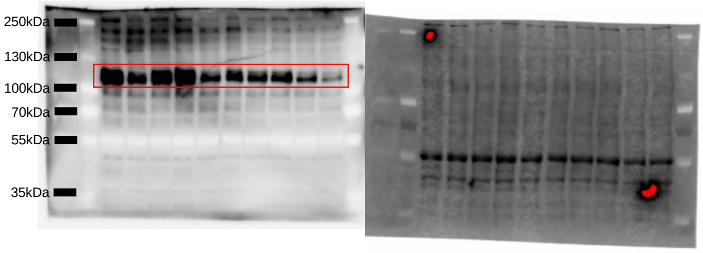


## 1.F

TrkB.FL

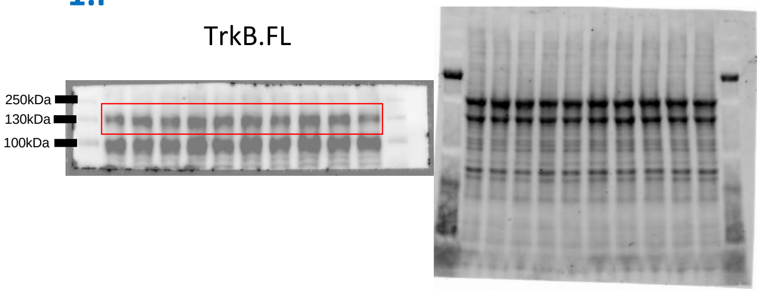


## 1.G

$P\text{-}Erk^{T202/T204}$

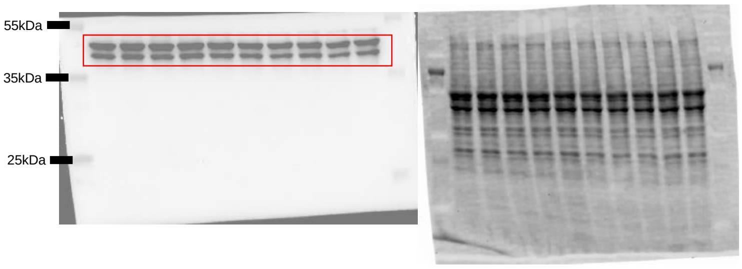


## 1.G

Erk

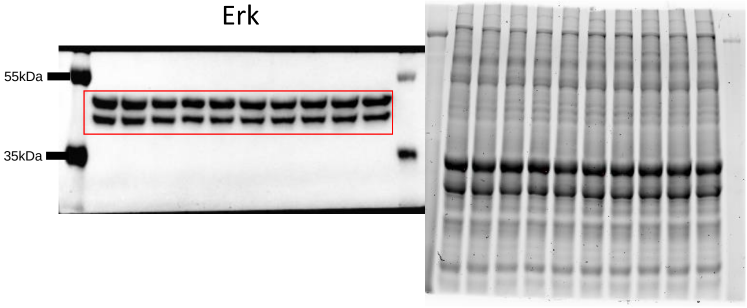


## 1.H

SYP

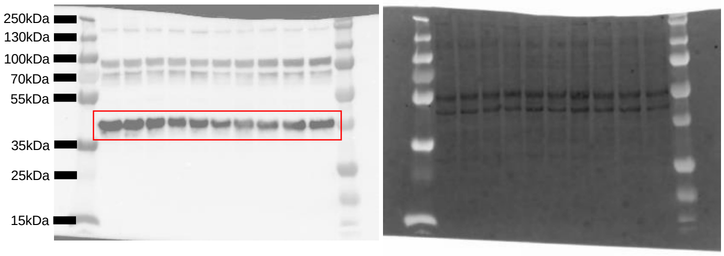


## 1.H

PSD95

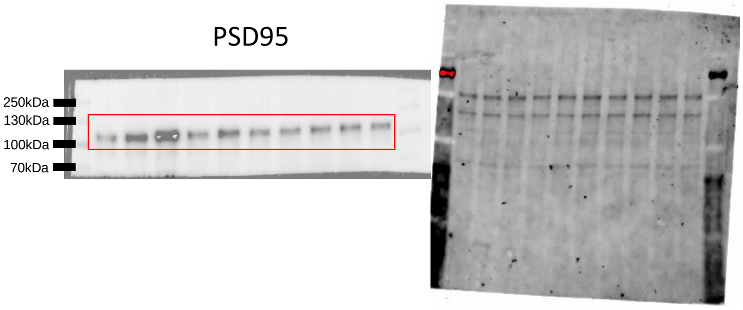


## 1.H

GAP43

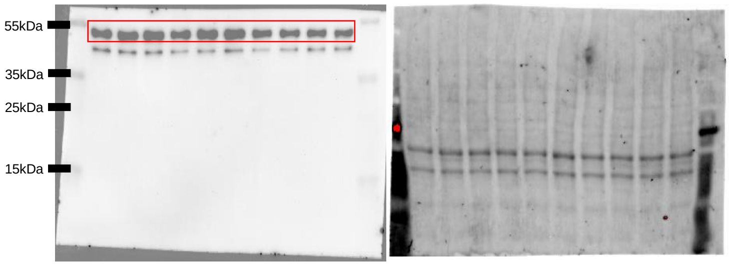

# Full unedited blots for Figure 2

## 2.A

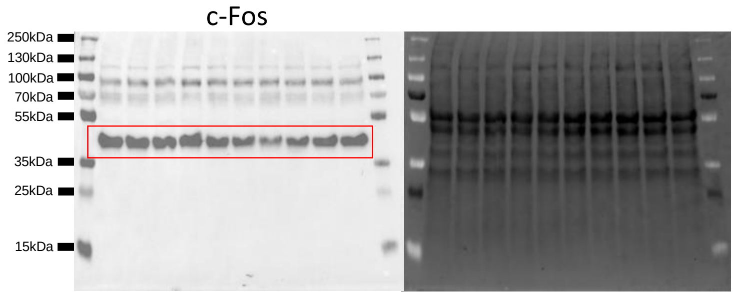


## 2.B

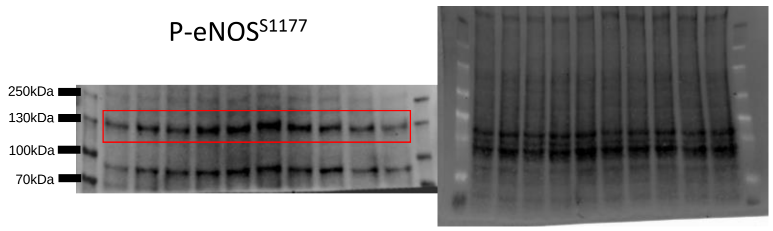


## 2.C

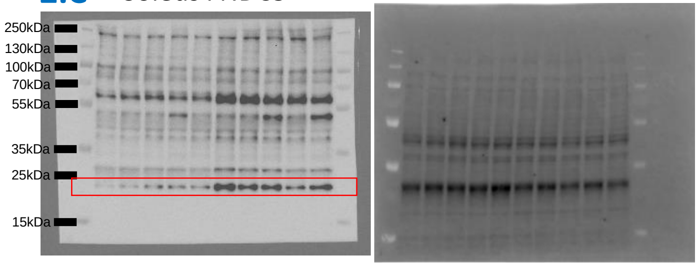


## 2.B

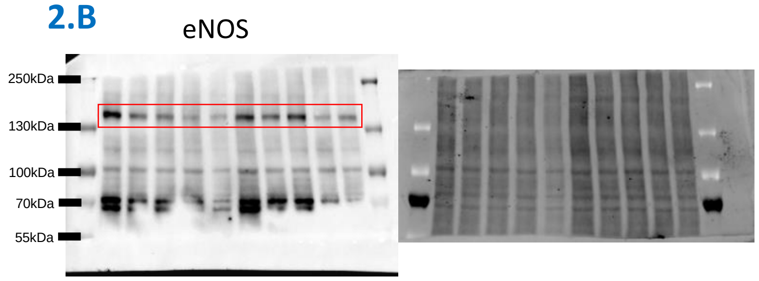


## 2.D

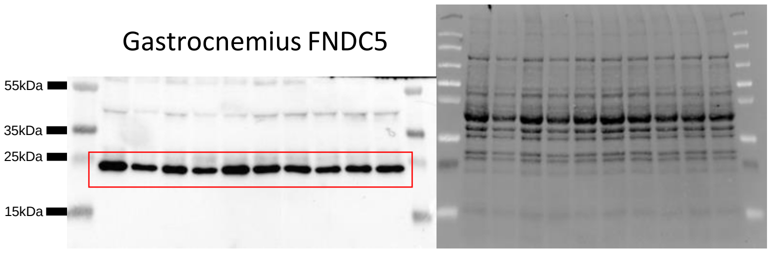


## 2.F

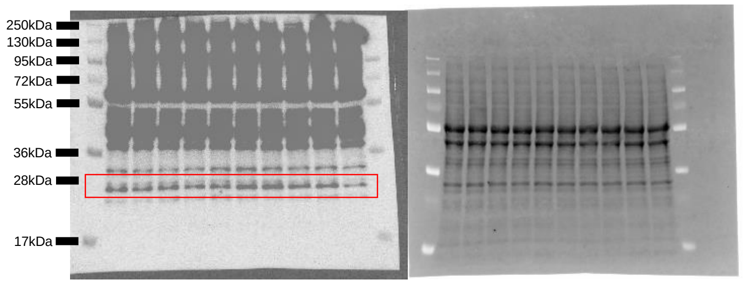


## 2.G

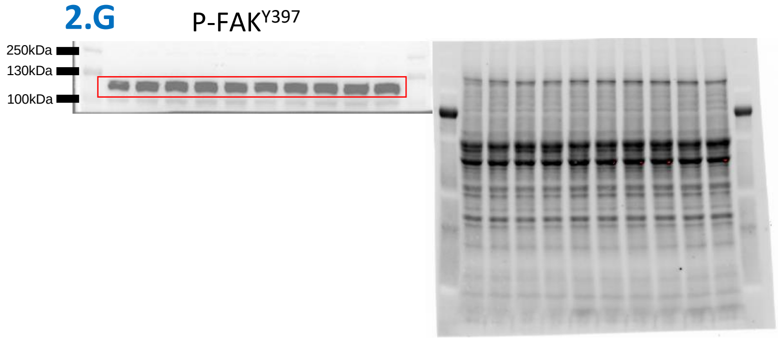

# Full unedited blots for Figure 4

## 4.A

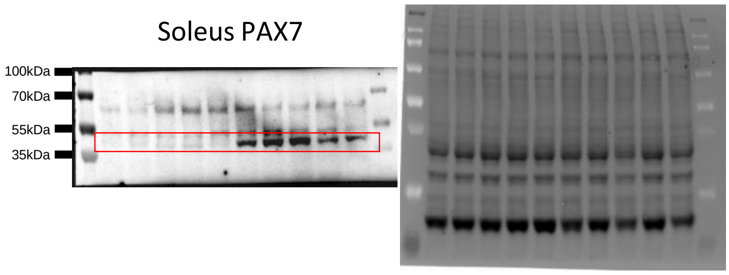


## 4.B

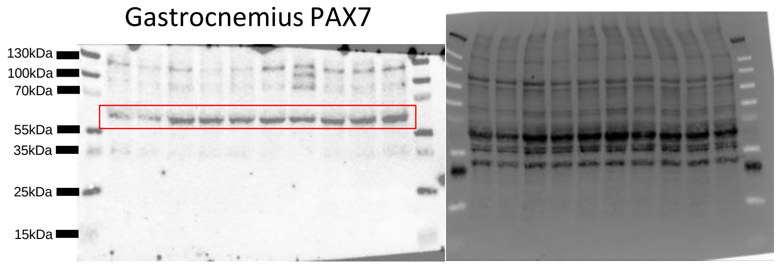


## 4.C

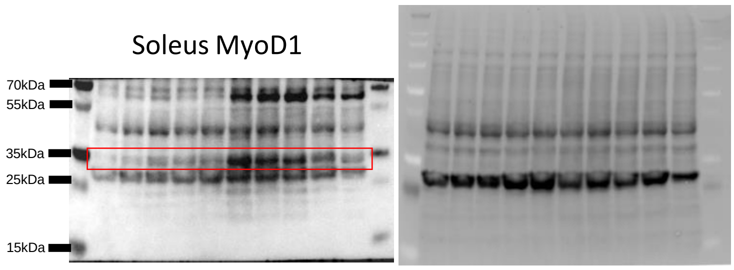


## 4.D

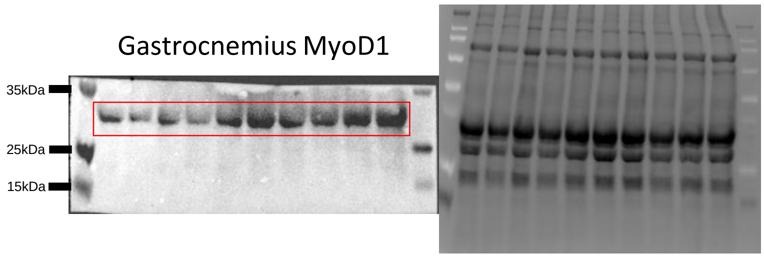

# Full unedited blots for Figure 5

## 5.C

Soleus IL-1β

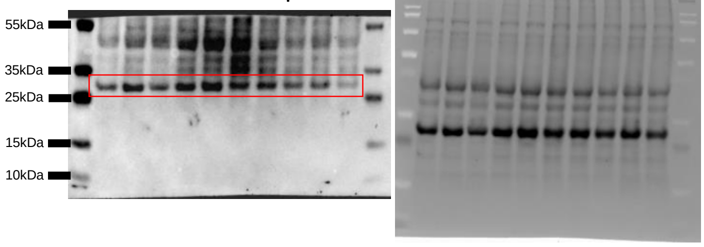


## 5.D

Gastrocnemius IL-1β

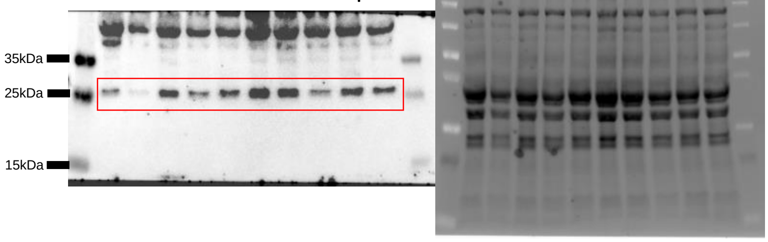


## 5.E

Brain IL-1β

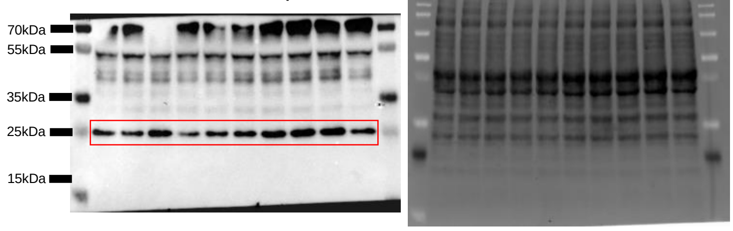


## 5.F

P-Glucocorticoid$^{S211}$ Receptor

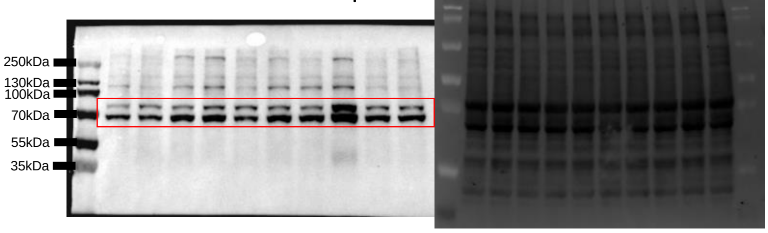

**Full unedited blots for Supplemental Figure 5**

## Supp.B

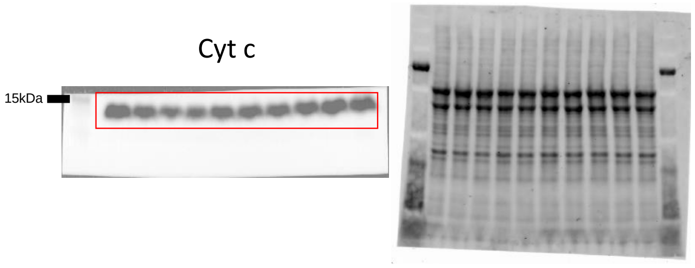


## Supp.C

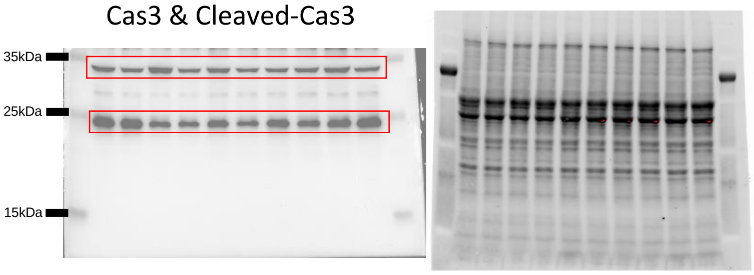


## Supp.D

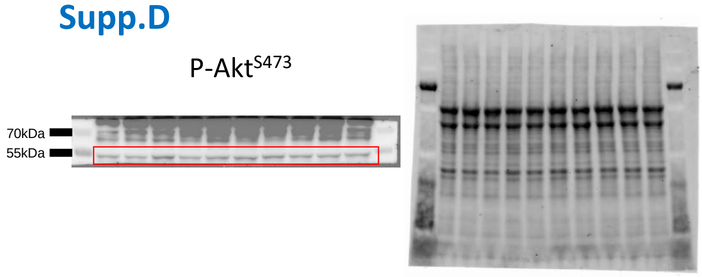


## Supp.D

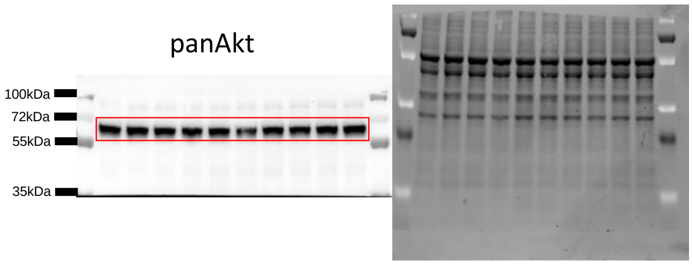


## Supp.F

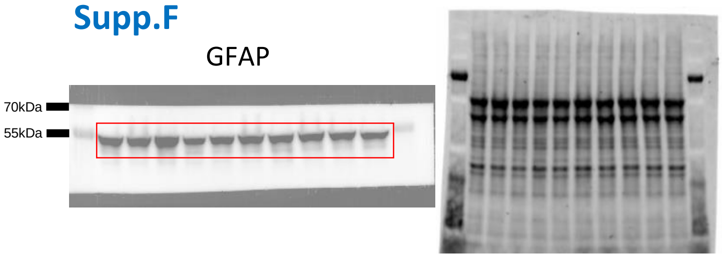


## Supp.G

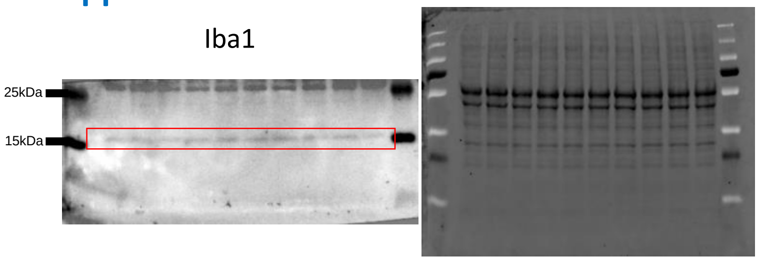


# <u>Reference of the protein ladder used for all Western blots:</u>

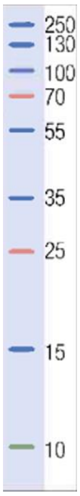


EUROMEDEX

**CERTIFICATE OF ANALYSIS**

**Prestained Protein Ladder Plus**

**#06P-0211 2x 250 µl**
(for 100 mini-gel applications 5µl per well or 50 large gel applications 10µl per well)
